# Missing millions: undercounting urbanization in India

**DOI:** 10.1007/s11111-019-00329-2

**Published:** 2019-12-05

**Authors:** Kyle Onda, Parmanand Sinha, Andrea E. Gaughan, Forrest R. Stevens, Nikhil Kaza

**Affiliations:** 1 Department of City & Regional Planning, University of North Carolina at Chapel Hill, Campus Box 3140, Chapel Hill, NC 27599 USA; 2 Department of Geography & Geosciences, University of Louisville, Lutz Hall, Louisville, KY 40292 USA

**Keywords:** Urbanization, Urban agglomerations, Urban-rural delineation, India, Gridded population data

## Abstract

The measurement and characterization of urbanization crucially depends upon defining what counts as urban. The government of India estimates that only 31% of the population is urban. We show that this is an artifact of the definition of urbanity and an underestimate of the level of urbanization in India. We use a random forest-based model to create a high-resolution (~ 100 m) population grid from district-level data available from the Indian Census for 2001 and 2011, a novel application of such methods to create temporally consistent population grids. We then apply a community-detection clustering algorithm to construct urban agglomerations for the entire country. Compared with the 2011 official statistics, we estimate 12% more of urban population, but find fewer mid-size cities. We also identify urban agglomerations that span jurisdictional boundaries across large portions of Kerala and the Gangetic Plain.

## Introduction

The global rate of urban transition has been immense in the past half century, with much of that transition and associated population growth occurring across parts of Asia (Ellis and Roberts 2015; Schneider et al. 2015). In 1960, India and China had similar urban population percentages of 18% and 16%, respectively (World Bank 2018). Yet by 2016, according to the World Bank statistics, while the Chinese urban population was at 54%, Indian urban population was at 33% suggesting very different developmental trajectories. In fact, the World Bank, based on Census of India statistics, estimates that urban India is growing at a declining rate (3.8% in the 1970s to 2.7% in the 1990s and 2000s, to 2.4% in the 2010s) (World Bank 2018). Widely varying estimates of such rates can be found from other sources. United Nations figures rely on national statistics that themselves are generated by a wide diversity of definitions of urban, leading to incomparable estimates of urban population and urbanization rates across countries (Uchida and Nelson 2010). In addition, a long-running debate exists in the literature about the relationship between urbanization of a country’s population and its economic growth (Fay and Opal 2000; Henderson 2003; Spence et al. 2009). While higher levels of urbanization are observed in countries with higher per-capita GDP, the rates of urbanization have little correlation to economic growth (Bloom et al. 2008; Chen et al. 2014).

Yet much of this literature presumes that urbanization levels, along with the GDP, are measured consistently and appropriately in different contexts (Satterthwaite 2007). Cross-country consistency in urban definitions is necessary for the design and study of urban policies that may vary by nation, such as the organization of public services or the allocation of development finance towards meeting international development goals (OECD 2012). For example, the Sustainable Development Goal 11, to “Make cities and human settlements inclusive, safe, resilient and sustainable”, is associated with a number of indicators and targets, the measured values of which can change substantially when applying different definitions and delineations of cities (Klopp and Petretta 2017).

Definitional differences are not just a matter for comparative convenience; they have both theoretical and policy implications. Studies of agglomeration economies and the determinants of urban economic growth in India often use districts as units of analysis due to a lack of availability of consistent boundaries for metropolitan areas, which would be a more appropriate unit for such research questions (e.g., Desmet et al. 2015; Duranton and Puga 2004; Ghani et al. 2016). This problem could potentially lead to misleading conclusions in cross-country comparative work. For example, Chauvin et al. (2017) conclude that India does not conform to spatial equilibrium, a central idea in urban economics, in a comparative analysis of India, Brazil, China, and the USA. In this study, districts were the unit of analysis for India, while units more analogous to Metropolitan Statistical Areas in the other three countries were used. In contrast, Hasan et al. (2017) find evidence of relatively low agglomeration economies in India based on town and city-level data, but do not account for how such towns may be part of larger metropolitan regions in their analysis.

From a governance standpoint, the delineation of urban areas has consequences for the spatial distribution of infrastructure provision and related institutional arrangements. Urban areas are seen as engines of economic development and infrastructural and resources are concentrated on them (Indian Planning Commission 2011). Even so, urban infrastructure investment is often assessed to be inadequate in India (Ahluwalia et al. 2014). Underestimating the existence of dense population clusters only exacerbates this problem by limiting the political attention, governance reform, and finance necessary to build and maintain appropriate levels of infrastructures such as intra-city transportation, water, sanitation, and health in dense, yet officially rural areas. Areas with high population density require qualitatively different types of infrastructure and necessitate different institutions to govern them than lower-density areas, regardless of whether they are administrated as urban or rural units (Rakodi and Lloyd-Jones 2002).

In India, rapid urbanization that was expected to follow economic liberalization policies starting in the 1990s was predicted to hollow out rural areas in favor of large urban areas such as Bengaluru due to migration based on economic opportunity. In part, these conclusions are drawn from undercounting urban areas and ignoring the large in situ urbanization happening over time. Denis et al. (2012) argue that close to two-fifths of the population live in urban settlements and 35% of the urbanites do live in small towns below 100,000 in population. More importantly, the patterns of urban settlements are different regionally, which also lead to regional developmental imbalances. For example, the less developed states of West Bengal and Bihar have substantially more dense settlements in the Denis et al. (2012) approach than the official estimates. Accordingly, Kundu (2011) argues that when optimistic rural-urban migration predictions were not realized, there were adverse consequences for urban livelihoods in smaller towns, which contribute little to national productivity and command little political attention. Indeed, initiatives such as the Jawaharal Nehru National Urban Renewal Mission (JNNURM), one of the largest infrastructure programs ever undertaken by the Government of India, allocated funds disproportionately to large urban areas and may have caused stagnation in smaller towns and their surrounding rural areas (Khan 2016).

Underbounding metropolitan areas has a related policy consequence when combined with India’s federalist governance structure. The 73rd and 74th Constitutional Amendments of 1993 devolved many planning and infrastructure provision responsibilities from state to local governments, including urban local bodies (ULBs) for officially urban areas and *gram panchayats* for officially rural areas. This devolution in some circumstances allowed local communities to organize appropriate institutions and infrastructure packages (Hutchings 2018). However, it also raises barriers for coordination between communities in the provision of some public goods or the management of shared common-pool resources. For example, the highly administratively fragmented Kochi urban area saw many JNNURM projects delayed or applications rejected due to competing priorities and conflict between the Kochi Municipal Corporation and surrounding ULBs and *gram panchayats* in the region (Kamath and Zachariah 2015). Such phenomena highlight the potential gains to be had from more regional planning structures that incorporate all neighboring clusters of high-density jurisdictions (whether administered by ULB or *panchayat*) into related infrastructure needs, as suggested by Mukhopadhyay et al. (2017).

The lack of a georeferenced and consistently delineated dataset also poses a problem for studying urban change over time. Official estimates put change in Indian urban population at 3.3% between 2001 and 2011, with 29.5% of this urban growth due to reclassification of rural areas into Census Towns by the Census of India, rather than expansion or densification of existing urban areas. This is higher than the growth in urban population attributable to migration (Pradhan 2013). However, the significance of these *invisible urban villages*, classified as urban by the Census but administered as rural areas, is not readily apparent due to the unavailability of appropriate georeferenced datasets. Since no fine-grained geographic and demographic data are readily available, researchers have to look for clues in various census tables to locate and measure the extent of such in situ urbanization. In this paper, we aim to make this urbanization visible, so that appropriate political and economic institutions can be fashioned to meet their governance needs.

## Background

There is no consistent definition of what constitutes an urban area around the world (Buettner 2015; Cohen 2006; Satterthwaite 2007). Previous efforts to define consistent, global definitions of urban area relied on daytime satellite images (Angel et al. 2011), nighttime lights (Zhou et al. 2011), functional integration (OECD 2012), and population density combined with travel times to the nearest large city (Uchida and Nelson 2010). Others have followed a more hierarchical definition of classifying the urban areas based on density, the proportion of the population living in different density clusters, population size, and contiguity characteristics (Dijkstra and Poelman 2014).

However, different statistical agencies use different definitions and, thus the measurement of urbanization and rates varies considerably from country to country. Some countries do not have specific criteria to delineate urban regions, instead preferring to list the urban areas with independent local governments. While many countries use a minimum population size (200–50,000), a few use minimum population density (~ 6.3 per ha) (Deuskar and Stewart 2016). India is one of the 16 countries that use criteria of economic activity (dominance of non-agricultural activity). India’s definition also has a gender dimension by counting only the type of jobs held by male workers. In particular, the Census of India defines urban areas as follows:

1. All places with a municipality, corporation, containment board, or notified town area committee, etc. (referred to as Statutory Towns)
2. All other places which satisfy all of the following criteria (referred to as Census Towns):
  - A minimum population of 5000
  - At least 75% of the male working population who work more than 6 months of the year engaged in non-agricultural work
  - A population density of at least 400 persons per square kilometer (4 persons per ha).

Despite the detailed definition, there exists considerable debate about the urban character of India and its evolution over time (Denis et al. 2012; Ganapati 2014; Sudhira and Gururaja 2012). For example, Denis et al. (2012) use contiguous built-up areas in India (with some leapfrogging) and assign the population of the Census-defined areas (not spatially demarcated). They then use a 10,000 person population threshold to classify urbanity for Indian cities. This definition and delineation allocate 100 million more people to urban areas compared with the Census of India 2011 estimates.

A variation of these approaches can be found in the works of Balk (2009) and McGranahan et al. (2007). Names and population estimates from the National Statistical Organizations (NSO) are merged with geographic coordinates for given administrative units from gazetteers. To define the urban extents, unlike Denis et al. (2012) which uses the daytime impervious surface, these approaches use nighttime lights, as a proxy for electrification, which is itself a proxy for urban service provision. The population from the NSO/gazetteer points within each urban extent is assigned to the polygon. Using urban extents from Balk (2009) to delineate large cities and including peri- and suburban areas that are within a certain distance from these large cities, Uchida and Nelson (2010) construct an agglomeration index as a characterization of the metropolitan region. Uchida and Nelson estimate the urban population of India to be between 42.9 and 51.9% compared with United Nation’s estimate of 27.7% (based on the Census of India 2000 estimates).

Each of these different definitions produces different urbanization estimates as well as extents and locations of urban agglomerations, with its own set of limitations. Using nighttime lights exclusively to define urban extent underestimates dense human settlements that are not yet electrified, or suffer from intermittent electrification provision or from light blooms (Abrahams et al. 2018; Small et al. 2005). In contrast, using exclusively daytime satellite imagery to delineate urban extents is constrained by weather conditions (e.g., cloud cover) and trade-offs between spatial and spectral resolutions. The inability of these methods to incorporate other types of data such as slope, hydrology, climatic zones, and other features such as infrastructure that are associated with human settlement patterns is critiqued by Uchida and Nelson (2010). Furthermore, relying on merging geographic coordinates to population data using place names is susceptible to significant error due to mis/multiple spellings and requires significant expert intervention. For example, about 1.8 million people in India were not assigned to a location in the Denis et al. (2012) approach. The contiguity criterion relied upon by Dijkstra and Poelman (2014) relies on a low spatial resolution (of 1 km) to delineate urban areas, resulting in fragmented and therefore small urban settlements, especially at the fringes of a city. In a different but still spatially compromised way, Uchida and Nelson’s agglomeration index merges spatially proximate but non-contiguous urban areas into one metropolitan area, changing the boundaries that can be used. Since their approach is to allow for cross-country comparisons of total urban population, the precise location and boundaries are less important.

We provide a methodology that allows us to define and delineate urban areas consistently across various jurisdictions. We propose a method called Metropolitan Agglomerations from Gridded Population Intensity Estimates (MAGPIE) that draws from the abovementioned approaches to characterize urban regions and their systems. We explicitly use density thresholds combined with size thresholds in a consistent fashion to distinguish between urban and rural settlements. We ignore the gender and economic activity thresholds that the Indian Census uses, for generalizability purposes. With relatively little human intervention, the proposed method produces an urban/rural delineation with an associated urbanization estimate similar to that of Indiapolis in short order. Because we rely upon gridded datasets, including remote sensing images, our conclusions are not bounded by jurisdictional vagaries. The other methods described in this section are also not limited by jurisdictions and allow for comparisons. However, they are limited by resolution and underlying covariates (Dijkstra and Poelman 2014) and imperfect separation of proximate urban areas (Uchida and Nelson 2010). MAGPIE addresses some of these limitations.

## Method

### Study area and data processing

Population counts were sourced from the Office of the Registrar General and Census Commissioner in India and population counts were linked to GIS administrative boundaries for each district (source: https://gadm.org/) creating a spatially explicit representation of population distribution at the census unit level. We do not include parts of Kashmir that do not have census data in our study region. We then modeled gridded population at the district level (*n* = 594) for the years 2001 and 2011, matching administrative boundaries for boundary and data consistency purposes between years, with 2001 as the base year. Fixed census units between years are important to enable a consistent estimation process across time (Gaughan et al. 2016). In doing so, we reduce the potential of under- or over-fitting the model due to heterogeneity in census unit size and associated average population densities.

We matched all covariate data for both years based on either temporally invariant or temporally explicit datasets. The land cover is based on GlobCover data, which is derived from the ENVISAT satellite mission’s MERIS (Medium Resolution Image Spectrometer) imagery. The land cover dataset has thirteen categories: cultivated terrestrial lands, woody/trees, shrubs, herbaceous, other terrestrial vegetation, aquatic vegetation, urban area, bare areas, water bodies, rural settlement, industrial area, built area, and no data. We also used digital elevation data and derived slope estimates from SRTM-based HydroSheds data (Lehner, Verdin, & Jarvis, 2013) and the DMSP-OLS (v.4) lights at nighttime series, obtained from NOAA’s National Geophysical Data Center(National Oceanic and Atmospheric Administration, n.d.). In addition, the Global Human Settlement Layer (GDAL/OGR Contributors, 2–19) with a spatial resolution of 38 m was collected from the European Commission Joint Research Centre (2014 beta version) for the years’ most coincident with 2001 and 2011. To best use the urban extent information, we created a distance-to-built-edge covariate, where distances inside the built land cover class boundary were negative and distances outside the edge were positive. We also used the WorldClim/BioClim 1950–2000 mean annual precipitation (BIO12) and mean annual temperature (BIO1) estimates (Hijmans, Cameron, Parra, Jones, & Jarvis, 2005). In addition to land cover, settlement, and associated raster datasets, we included geospatial data that was correlated with human population presence on the landscape, such as protected area delineations (UNEP-WCMC, 2010), networks of roads, and waterways; large water bodies; and infrastructure-related features and settlement or populated locations from open street map 2017. All these covariate data employed in the modeling process are summarized in Table 1. These covariates were all summarized to the district polygon level as the average value within each polygon. All datasets were resampled using nearest neighbor to match the same resolution to a square pixel resolution of 8.33 × 10^−4^ degrees (approximately 100 m at the equator) and projected into UTM 44N projection prior to analysis. All covariates were prepared in ArcGIS (ESRI 2016) and Python programming language (version 2.7) (Python Software Foundation 2013).

**Table 1:** Covariates used in gridded population modeling process

Variable name(s)	Source and nominal resolution
District Census Population, 2001, 2011	Open Government Data (OGD) Platform India, district level
Temporally explicit covariates	
Land Cover, 2000, 2010	GlobCover, 300 m
Global Human Settlement Layer, 2000, 2012	ECJRC, 38 m (Pesaresi et al., 2013)
Lights at night, 2001, 2011	DMSP-OLS-derived (National Oceanic and Atmospheric Administration, n.d.)
Common covariates	
Mean temperature, 1950–2000	WorldClim/BioClim (BIO1) (Hijmans et al., 2005)
Mean precipitation, 1950–2000	WorldClim/BioClim (BIO12) (Hijmans et al., 2005)
Sanctuaries, National parks, Game Reserves, World Heritage Sites	World Database on Protected Areas September, 2012, UNEP (IUCN, UNEP-WCMC, 2010)
Elevation	USGS HydroSHEDS (Lehner et al., 2013)
Derived Slope	USGS HydroSHEDS (Lehner et al., 2013)
Distance to infrastructures	Open Street Map, 2017–05
Distance to places	Open Street Map, 2017–05
Distance to road networks	Open Street Map, 2017–05
Distance to waterbodies	Open Street Map, 2017–05

### Gridded population intensity estimates

We generated gridded population intensity estimates (GPIE) using the methods described by Stevens et al. (2015) to disaggregate the census population for 2001 and 2011. We used grid cells with a resolution of 3 arc sec (approximately 100 m at the equator). We used a random forest (RF) statistical model (Breiman 2001) to generate a population prediction density layer, in conjunction with a dasymetric redistribution of population counts (Stevens et al. 2015) to produce final gridded population outputs at approximately 100 × 100 m grid cells. For the Indian subcontinent, this represents approximately 395 million pixels of land that population is allocated to. The RF statistical model provides a non-parametric platform coupled with an ensemble machine-learning technique for classification or prediction purposes (Breiman 2001). The RF method relies on the use of bagging and random selection of covariates across numerous classification and regression trees (Lung et al. 2013).

**Fig. 1:**
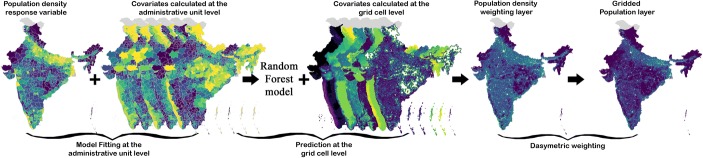
Schematic representation of the dasymetric gridded population modeling process

For our purposes, we use census counts at the district level and covariate aggregation values for each census unit to create a RF model to predict log population density (Lung et al. 2013). In this method, the dasymetric redistribution weight is produced as a function of different covariates representing the individual covariates such as lights-at-night, slope, elevation, and proximity to land-use types. The resulting RF is used to predict a country-wide, pixel-level map of log population densities that provides a weighting layer for a dasymetric redistribution scheme (Mennis 2003) to redistribute population counts within each unit to the target cells (Stevens et al. 2015). Figure 1 portrays the schematic process involved in creating the dasymetric weighting layer. This dasymetric disaggregation then unevenly allocates the district-level population to underlying raster respecting the protected and uninhabitable areas. The result of this process is a 100 × 100 m grid cell (1 ha) resolution population map for 2001 and 2011. RF model fitting at the administrative unit level and prediction at the grid cell level were both performed in the R statistical environment (R Development Core Team, 2017) using the randomForest package (Liaw and Wiener 2002). Predicted range of population datasets from random forest models is sensitive to the scale of the training dataset and using a coarse dataset could lead to a small range in dasymetric weighting surface. As a result, with coarse census data, a less heterogeneous population density will be observed with fewer extremes. We also note a disconnection between the level of support between the model estimated for administrative units and the scale of the predictions from the model used to disaggregate census data. However, while no assumptions are placed on the linearity or interactions present in relating ancillary data to population density (a feature of random forest modeling), we assume that the process resulting in those estimated associations at an aggregate level are, on the whole, representative of the process relating covariates to population density at the finer, gridded scale. In the absence of data on population densities at the finer scale of interest, of which we have none to estimate the model with or validate against across time, output based on this assumption has consistently shown to perform better than less complex or less informed disaggregation techniques (Stevens et al. 2015; Gaughan et al. 2016; Nieves, et al. 2017). Despite the “ecological fallacy” inherent to this change-of-support (Gelfland, et al. 2001; Holt, et al. 1996) and likely biased outcome at the pixel level, the approach still manages to achieve comparable results to bottom-up modeling using fine-scale model estimates (e.g., Engstrom, et al. 2019).

### Metropolitan agglomerations

**Fig. 2:**
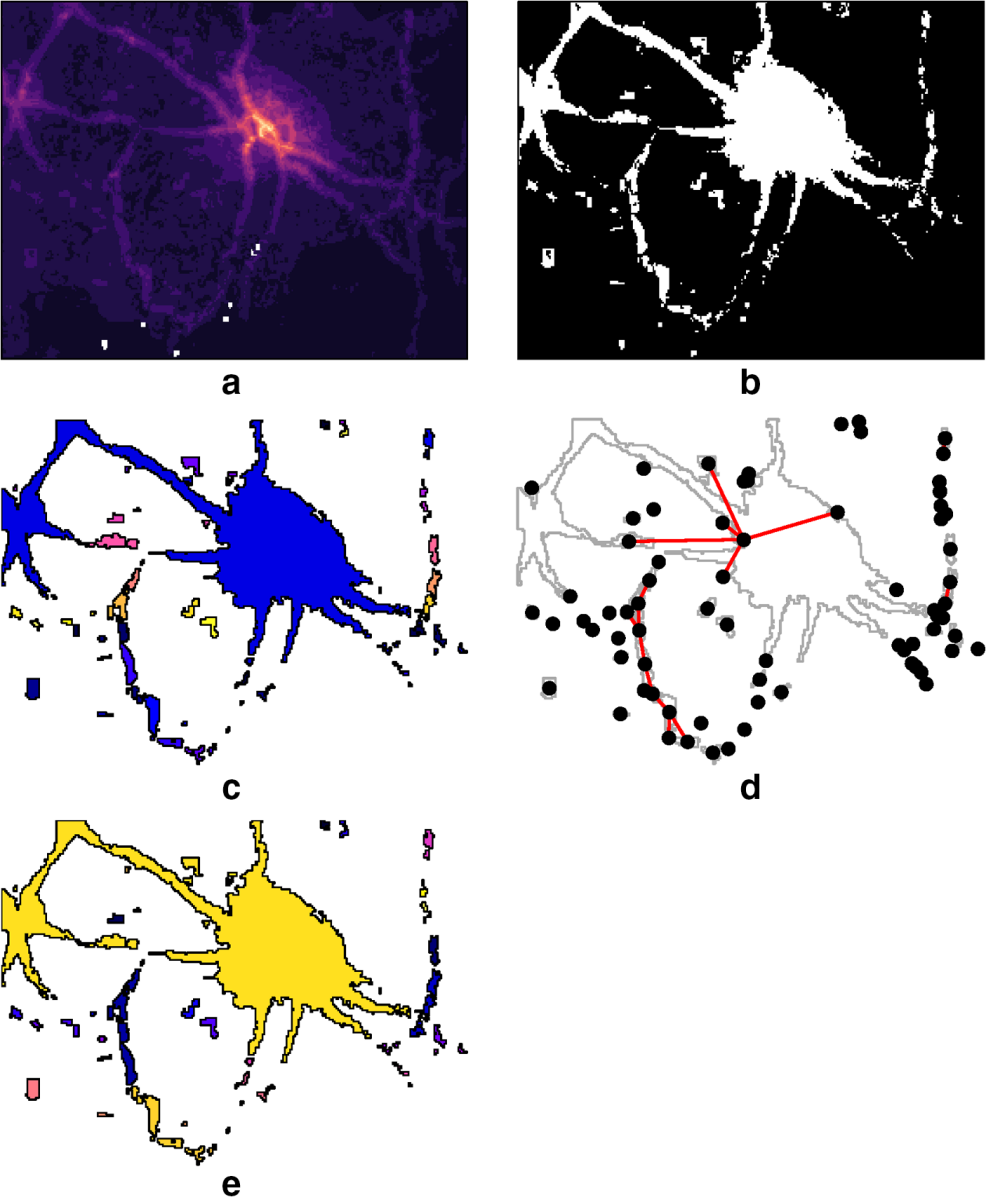
Various stages of defining the urban area boundary in MAGPIE. **a** Input population intensity estimates. **b** Urban areas based on density threshold. **c** Removal of holes and polygonization based on contiguity constraint. **d** Construction of graph based on distance threshold to account for non-contiguous polygons. **e** Construction of clusters based on eigenvector community-detection technique

We define urban areas in metropolitan agglomerations (MA) using a three step process (see Fig. 2). Based on the GPIE, we first select all cells that are outputs that are above a certain density threshold. We use 7.5 persons per ha as a density threshold and experiment with 5 and 10 persons per ha to test the sensitivity of this threshold. Note that all of these are above the 4 and 3 person per ha thresholds used by Census of India and Dijkstra and Poelman (2014), respectively.^1^ We use contiguity of these densely populated cells to construct clusters of urbanized areas using a region grouping algorithm from Geospatial Data Abstraction Library (GDAL/OGR contributors 2019). Holes within each of the polygons are removed. In other words, unpopulated areas that are completely circled by urban areas such as hills, parks, and lakes would be considered to be within the boundary of the urban area. This removal of holes adds 9% more to the urbanized area than otherwise and only has marginal effect on the urban population estimates (~ 2.8%). Because of noise associated with GPIE, we removed areas that are below 2 ha area from consideration. The 2 ha are approximately two contiguous cells that are not adjacent to any other selected cells. We experimented with different thresholds and selected 2 ha as the areal threshold that produces urban population estimates less than 90%.

However, contiguity is an insufficient criterion to delineate metropolitan areas, as they are usually fragmented at the edges. To determine how these constellations of fragments relate to one another and to larger urban areas, we turn to the community-detection algorithms borrowed from network science (Comber et al. 2012; He et al. 2019). We then construct a graph from these polygons with each polygon as a node with the vertex set *V*(*G*). Two nodes are connected with an edge if the distance between their boundaries is below a distance threshold of 150 m. This is a distance that is roughly the diagonal of the cell and approximates queen contiguity criterion with one cell skipped over. We then find communities within the components of the graph *G* using the leading non-negative eigenvector of the modularity matrix of the graph (Newman, 2006). Community-detection techniques allow us to partition the vertices of the graph into groups, where the connections within the groups are denser than the connections between groups. The intuition is that if multiple urban clusters are close to one another, they should be treated a coherent entity. This also allows us to avoid identifying tendril-or dumbbell-like urban patterns, unless they are explicitly contiguous. We then combine the polygons represented by the vertices that are part of a community into a single metropolitan agglomeration. This analysis is done using raster (Hijmans 2017), spdep (Bivand and Piras 2015), and igraph (Csardi and Nepusz 2006) packages in R.

## Results

**Fig. 3:**
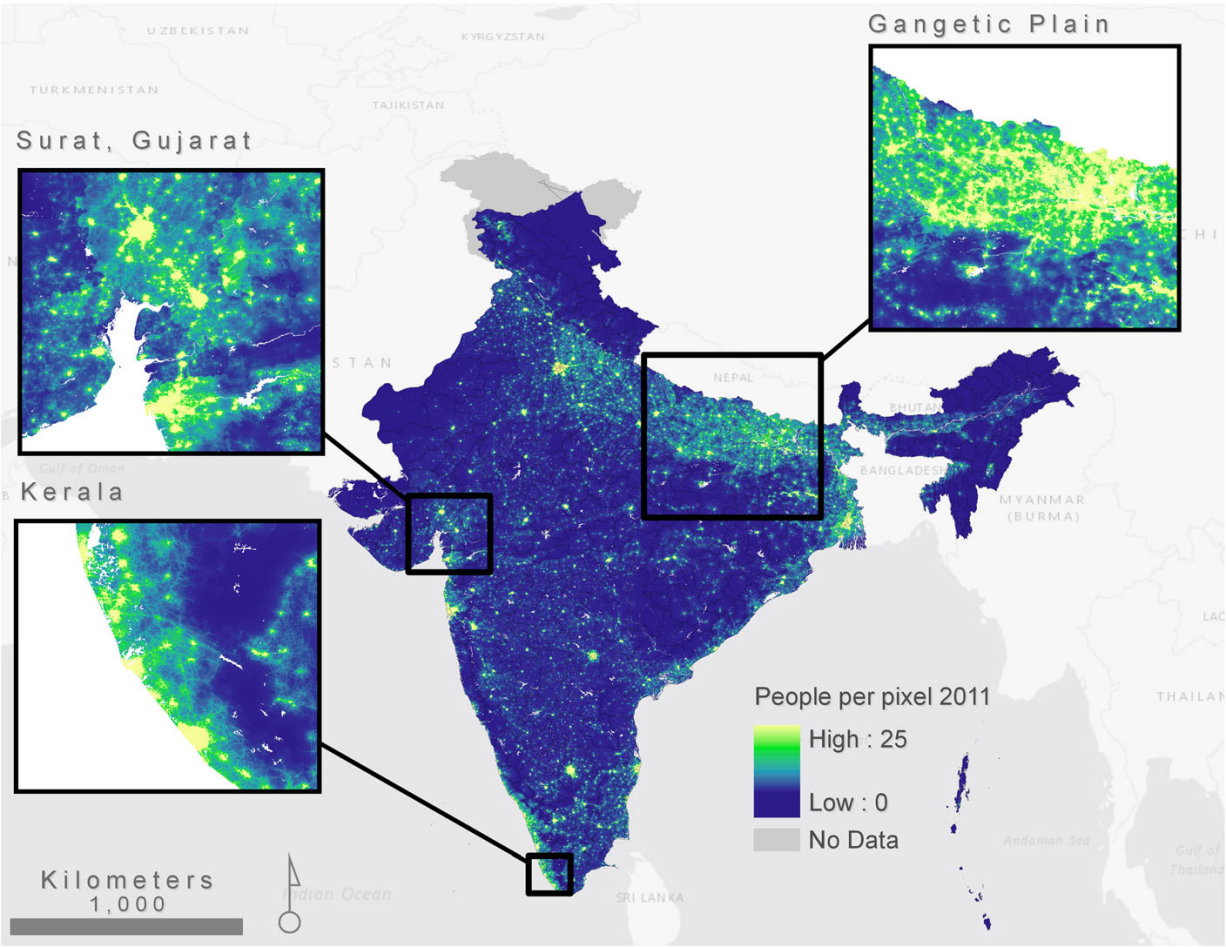
Gridded Population Intensity Estimates for India (2011). Maximum value is restricted to 25 for visualization purposes

In order to measure the prediction error of the random forest model, we estimate the out-of-bag (OOB) error from 37% samples with 500 trees. The OOB is an error estimate calculated during the RF model fitting and is based on averaging all mean squared errors. It provides a robust and unbiased measurement of the prediction accuracy of the RF model (Breiman 2001) and informs the accuracy of the final gridded population datasets produced using the RF-based approach (Gaughan et al. 2016; Stevens et al. 2015). The pseudo-r-square value for training model based on mean population density at district scale is 0.88 and 0.87 for 2001 and 2011, respectively. The median values of predicted population counts are 1.4 to 1.6 persons per pixels (see Fig. 3). To assess the final accuracy of the GPIE estimates, we matched 500 randomly selected village/town boundaries (level 4 administrative units, available from Bhuvan, a high-resolution web mapping service focused on the region of India (National Remote Sensing Center 2019)) with the census population counts using name of the village/town and district as an identifier. We aggregated the GPIE results to the village/town boundaries after correcting for boundary errors and projection issues. The correlation coefficient is 0.86 between census counts and GPIE results providing confidence in the spatial representation of gridded population outputs.

We report our estimates for metropolitan agglomerations with each of the three density thresholds (5, 7.5, and 10 persons per hectare) with a 150-m distance threshold.^2^ We compare our results with those of three other urbanization estimates; the Census of India, Indiapolis (Denis et al. 2012) and GHS-POP/GHS-SMOD. We produce the GHS-POP/GHS-SMOD estimate by aggregating the 2015 estimate of the 2019 version of the Global Human Settlement population grid (GHS-POP) (Schiavina et al. 2019) with the urban settlements of the 2019 version of the GHS settlement model grid (GHS-SMOD) (Pesaresi et al. 2019). To create the settlement clusters from GHS-SMOD, we combine raster cells classified to be in the “urban domain” that are contiguous at the edges (and not only the corners) into polygons representing discrete contiguous settlements. It should be noted that these are the best available estimates at the time of publication and that they are subject to continual updates.

We divide our results into the following sections: (1) characterization of the location and type of urbanization for 2011, (2) comparison of our method with the Census of India, Indiapolis and GHS-POP/GHS-SMOD estimates of urbanization and urban hierarchy for 2011; and (3) comparison of estimates for urbanization rates between 2001 and 2011 with the Census and Indiapolis.

### Patterns of urban settlements in 2011

**Fig. 4:**
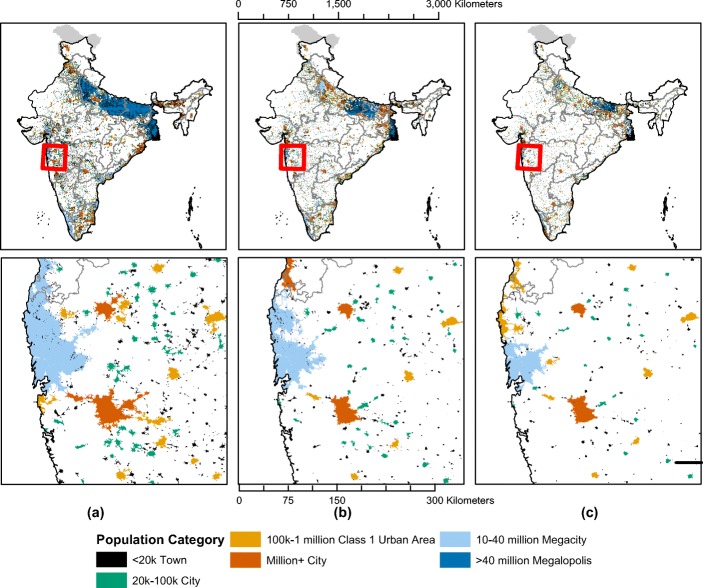
Spatial extent of urbanization according to MAGPIE. Top row for the entire map of India, bottom row inset for the detail of smaller areas. Estimated with a minimum density threshold of (**a**) 5 person/ha., (**b**) 7.5 person/ha. (**c**), and 10 person/ha

**Fig. 5:**
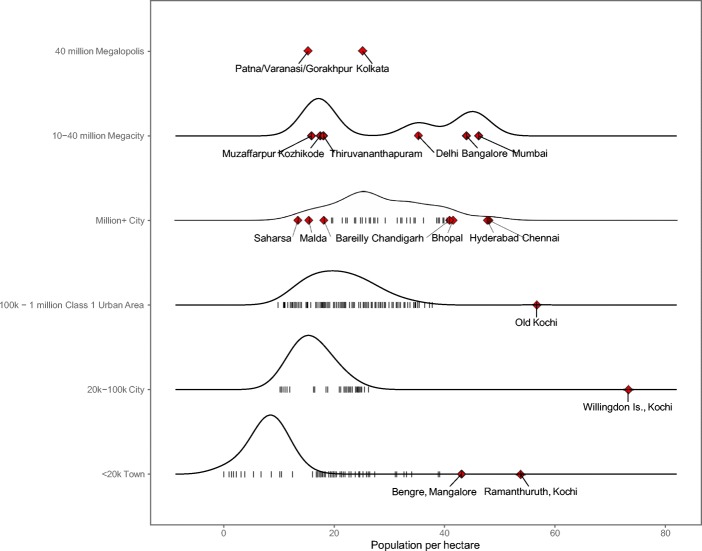
Relationships among MAGPIE estimates of population and population density in India in 2011 (7.5 pp./ha threshold). Vertical lines represent individual urban agglomerations, positioned on the *x*-axis according to population density (population per hectare) and on the *y*-axis according to population size category. Red diamonds represent particularly high- or low-density urban agglomerations of interest

Figure 4 shows the spatial extent of urbanization and the sensitivity to the minimum density thresholds. Lowering the density threshold results in larger solitary cities and the coalescence of cities into larger and more populous agglomerations (see Fig. 4a, b). Regions where estimated urbanized areas greatly increase in size when the density threshold changes generally indicate large areas of a relatively uniform population density in between denser urban centers. These regions are easily identifiable as the large contiguous megalopolises with greater than 40 million people including the extended agglomeration of Delhi and western Uttar Pradesh, the Gangetic plain through eastern Uttar Pradesh and Bihar (labeled for urban centers Patna, Varanasi, and Gorakhpur in the top row of Fig. 5), and most of West Bengal (labeled Kolkata in Fig. 5). Kerala exhibits a similar pattern of contiguous medium-density settlement, but without the numerous dense urban centers in between that would push the total population above 40 million. These regions (labeled urban centers Kozhikode and Thiruvananthapuram in Fig. 5) have comparable population sizes to large cities like Mumbai and Bangalore but much less dense, suggesting coalescent urbanization that knits together many villages, towns, and cities.

**Fig. 6:**
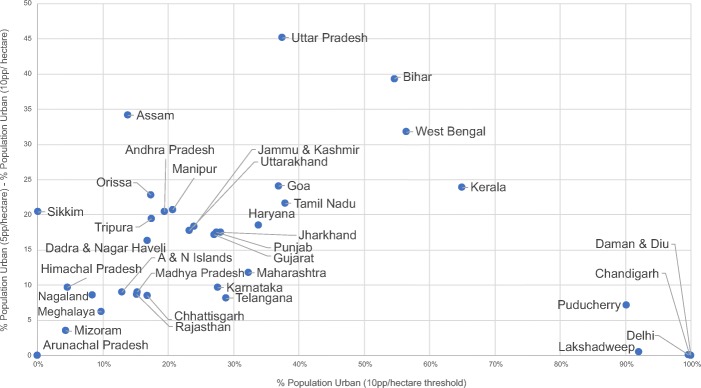
Characterizing the type of urbanization in 2011 by State and Union Territory according to MAGPIE. Bottom right corner indicates that regions that are highly urbanized and the density thresholds matter little for urban population counts. Top left corner are regions where density thresholds matter greatly for the counts

Outside of these regions, the pattern of urbanization is different. The relative lack of change in the size of urban areas of at least 100,000 in population when the density thresholds are changed indicates that populations are more highly concentrated in urban areas. Estimates of the urban population in these regions rise as the density threshold falls, but this is due to a combination of two reasons: (1) higher numbers of distinct settlements are counted as urban and (2) the periphery of urban areas is now included within the boundary of an existing urban area. An example of this kind of urbanization pattern is throughout the state of Maharashtra, where areas of high population density are concentrated around Mumbai, Pune, Nagpur, and several cities between 100,000 and 1000,000 in population and new agglomerations are not created by lowering the density threshold (see insets in bottom row in Fig. 4). Metropolitan agglomerations such as Chennai, Bangalore, and Hyderabad also follow this pattern, being the densest population centers in India, as they are not connected to other large cities and so are not as populous as the megalopolises (see third row of Fig. 5). Another way to understand the pattern of urbanization is to see how the density thresholds affect the proportion of people that are considered urban in each state. Uttar Pradesh is dramatically affected by the threshold, suggesting a 45-percentage point difference; changing the density threshold from 5 to 10 decreases the urban population by 45 percentage points (see Fig. 6). A similar, but less dramatic, effect is observed in Bihar, Assam, and West Bengal. In contrast, due to high densities, many union territories and the Delhi region are not affected by the threshold.

### Comparison with other estimates

Different methods produce vastly different estimates of urbanization in India (see Table 2). The categories in Table 2 are based on various size thresholds used by the Census of India in publications of the populations of urban areas (Registrar General and Census Commissioner of India 2011). Urban areas with less than 5000 people do not meet the qualifications to be counted as a census towns, so any tabulated are statutory towns in the Indian census and there is no published count of the ones that are not considered part of larger urban areas. The next category is urban areas with between 5000 and 20,000 people but there is similarly no specific tabulation of them. “Urban agglomerations” are composed of combinations of towns, cities, and “out growths” with a combined population of at least 20,000 in 2001. “Class 1 urban agglomerations” have at least 100,000 people, “million plus cities” at least 1 million, and “megacities” at least 10 million people. Thus, based on these thresholds, we tabulate counts and populations of urban areas based on population size breaks of 5000; 20,000; 100,000; 1 million; and 10 million. Our methods in some cases produced large agglomerations with more than twice the population of what are normally considered India’s largest cities of Delhi, Mumbai, and Kolkata. We categorize these agglomerations, with populations greater than 40 million, as “megalopolises.”

**Table 2:** Comparison of counts of urban agglomerations and population estimates in 2011 (2015 for GHSL) for different size categories by the Census of India, Indiapolis, GHS-SMOD, and MAGPIE at 10 pp/ha, 7.5 pp/ha and 5 pp/ha population density thresholds

	Census	Indiapolis	GHS-POP and SMOD	MAGPIE		
Counts				10 pp./ ha	7.5 pp./ ha	5 pp./ha
1. Megalopolis (> 40 million)				1	2	2
2. Megacity (10–40 million)	3	4	5	5	6	6
3. Million+ urban area (1–10 million)	50	47	72	47	46	30
4. Class 1 urban area (100k–1 million)	415	495	1556	330	329	275
5. 20–100k town	1846	2866	5275	782	769	739
6. 5–20k town	NA	4272	15,182	1000	1018	983
7. < 5k town	NA	Not considered urban	194	10,269	20,804	32,693
Population						
1. Megalopolis (> 40 million)				40,251,032	116,483,274	364,555,198
2. Megacity (10–40 million)	48,802,734	73,894,637	104,736,702	105,197,661	122,513,999	113,912,852
3. Million+ urban area (1–10 million)	111,813,450	116,694,703	179,271,190	127,104,331	136,097,571	95,663,062
4. Class 1 urban area (100k–1 million)	104,293,727	117,489,953	358,730,669	87,419,632	91,539,353	70,548,571
5. 20–100k town	74,112,244	110,112,901	226,556,092	35,992,827	34,445,915	32,843,228
6. 5–20k town	38,098,826	58,464,875	141,688,151	10,634,852	10,799,146	10,115,771
7. < 5k town			462,143	4,919,263	6,886,963	7,548,863
Total	377,120,981	476,657,069	1,011,444,947	411,519,597	518,766,222	695,187,545
% Urban	31%	39%	77%	34%	43%	57%

The 2019 GHS-POP/GHS-SMOD estimates, based on the method of Dijkstra and Poelman (2014), place urbanization (in 2015) in India at 77%, which is much higher than MAGPIE, Indiapolis, and the Census of India. This is likely because the underlying dasymetric population disaggregation only uses one binary covariate, the GHSL built-up area indicator (Corbane et al. 2018; Florczyk et al. 2019). Combined with the relatively coarse 1-km spatial resolution, the effect is to consider a large proportion of square kilometer grid cells in India with any built-up area as urban. We find this to be an implausibly high estimate of urbanization in India.

MAGPIE tends to produce different urban hierarchies than the Census as well as the Indiapolis project in three respects. First, our method produces large numbers of isolated, small urban areas with less than 5000 people, although all of these towns together only amount to 5–8 million people. These settlements are generally not considered urban by the Census and are categorically not considered urban by Indiapolis.

Second, MAGPIE tends to consider large areas of relatively high population density (though not necessarily concentrated around traditional core cities) as urban. This results in the reallocation of the Indian population from small towns as well as areas the Census considers rural into larger urban areas with more than 10 million people. For instance, our most conservative threshold combination of 10 persons/ha with a distance threshold between settlements of 150 m produces two large urban agglomerations of roughly 30 million people each in Bihar and considers almost all of the coast of the state of Kerala as one contiguous agglomeration with over 40 million people. Our method’s characterization of urbanization in Kerala is similar to that of Indiapolis.

Third, our method tends to agglomerate populous municipalities with dense networks of smaller settlements in between them into larger agglomerations, reducing the number of mid-size cities and increasing the number of megacities relative to the census. For instance, our method folds many areas that the Census and Indiapolis consider cities with populations between 100,000 and 10 million into larger megacities, while combining almost all of Uttar Pradesh, Bihar, and West Bengal into megalopolises.

**Fig. 7:**
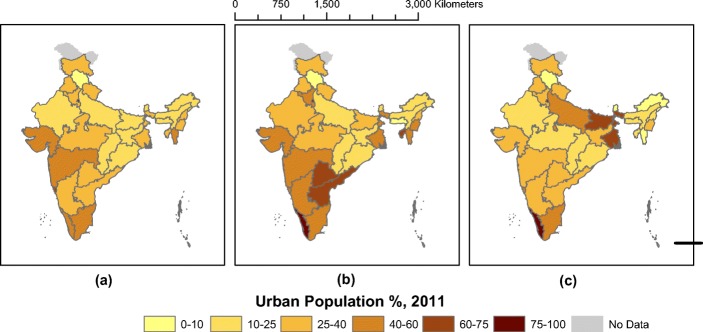
Comparison of the 2011 urbanization level by state. **a** Census of India. **b** Indiapolis. **c** MAGPIE (7.5 persons/ha)

MAGPIE also results in different urbanization estimates at the state level. Figure 7 summarizes the urbanization rates of the Indian states as calculated by (a) the Census of India, (b) Indiapolis, and (c) MAGPIE estimates with 7.5 persons/ha threshold. MAGPIE generally estimates lower urbanization rates for each state than the Census. The major exceptions are Kerala, Bihar, Uttar Pradesh, and West Bengal, where we estimate much higher urbanization rates than the Census. We characterize urbanization in Kerala similarly to Indiapolis, although we estimate a much higher degree of urbanization in Bihar, Uttar Pradesh, and West Bengal than does Indiapolis. By contrast, MAGPIE tends to estimate lower urbanization than the Census or Indiapolis in mountainous states such as Mizoram, Nagaland, and Sikkim, as well as in Gujarat and Maharashtra. However, since Bihar, Uttar Pradesh, and West Bengal are very populous states, our estimates of higher urbanization in these states outweigh our lower estimates in the other states to create a higher estimate of national urbanization.

The overall effect is a higher proportion of the Indian population being urban than in the official figures. For 2011, at the 7.5 pp./ha threshold, we estimate overall urbanization at 43% (compared with the Census estimate of 31%). This amounts to a difference of 140 million people from the Census. This also suggests a much different urban hierarchy than the Census implies, with much larger proportions of the Indian population allocated into urban areas with greater than 10 million people and relatively fewer people in cities with less than 100,000 people.

### Temporal change: urbanization between 2001 and 2011

**Fig. 8:**
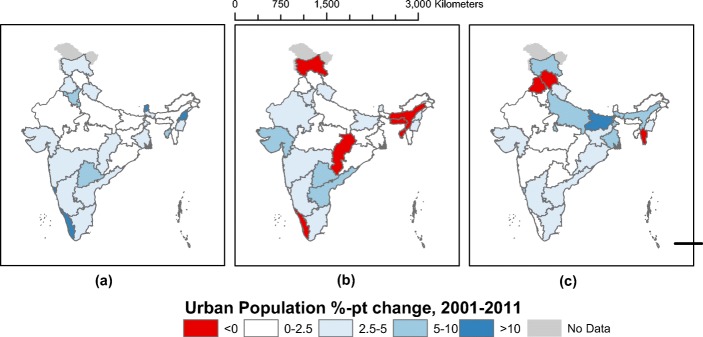
Change in urbanization by state, 2001–2011. **a** Indian Census. **b** Indiapolis. **c** MAGPIE estimates

We estimate a change of 4.7 percentage points in the proportion of urban population in India between 2001 and 2011 (see Table 3). This is not significantly different from other estimates such as Census (3.3 points) and Indiapolis (2.4 points). However, there is significant heterogeneity in estimates of rates of urbanization at the scale of the state. While the Census estimates significant urbanization in the south India, it undercounts the rate of urbanization in Gujarat relative to Indiapolis (see Fig. 8). According to our estimates, while southern Indian states have experienced higher urbanization rates, they are dwarfed by the urbanization rates in Uttar Pradesh, Bihar, and West Bengal. While these states have not traditionally been at the forefront of urbanization, they seem to be densifying quite rapidly in a way that is not being captured by the Census estimates. However, unlike Indiapolis, we do not estimate a marginal decline in the urbanization in the heavily urbanized state of Kerala (see Fig. 8 and Table 3).

**Table 3:** Comparison of estimates of change in urbanization between 2001 and 2011 at the state level, as estimated by the Census of India, Indiapolis, and MAGPIE

	Census			Indiapolis			MAGPIE (7.5 persons/ha)		
State	2001	2011	% point difference (%)	2001	2011	% -point difference (%)	2001	2011	% -point difference (%)
Andaman & Nicobar Islands	33%	38%	5%	30%	33%	3%	15%	16%	1%
Andhra Pradesh	24%	30%	5%	36%	41%	5%	23%	26%	3%
Arunachal Pradesh	21%	23%	2%	16%	16%	0%	0%	0%	0%
Assam	13%	14%	1%	21%	22%	0%	16%	24%	8%
Bihar	10%	11%	1%	31%	36%	5%	63%	74%	11%
Chandigarh	90%	97%	7%	99%	99%	− 1%	100%	100%	0%
Chhattisgarh	20%	23%	3%	21%	21%	− -1%	18%	20%	2%
Dadra and Nagar Haveli	23%	47%	24%	44%	53%	9%	12%	26%	14%
Daman and Diu	36%	75%	39%	87%	95%	8%	121%	115%	− -6%
Delhi	93%	98%	4%	97%	97%	1%	100%	100%	0%
Goa	50%	62%	12%	57%	57%	1%	40%	45%	6%
Gujarat	37%	43%	5%	43%	53%	10%	30%	33%	3%
Haryana	29%	35%	6%	38%	43%	5%	37%	39%	2%
Himachal Pradesh	10%	10%	0%	8%	9%	0%	8%	7%	−1
Jammu and Kashmir	25%	27%	3%	31%	31%	− 1%	23%	31%	7%
Jharkhand	22%	24%	2%	25%	25%	0%	28%	34%	5%
Karnataka	34%	39%	5%	38%	43%	4%	26%	31%	4%
Kerala	26%r	48%	22%	97%	96%	− 1%	77%	78%	1%
Lakshadweep	44%	78%	34%	34%	51%	17%	84%	93%	9%
Madhya Pradesh	26%	28%	1%	26%	27%	1%	17%	18%	1%
Maharashtra	42%	45%	3%	48%	51%	3%	35%	36%	1%
Manipur	25%	29%	4%	47%	52%	5%	24%	27%	3%
Meghalaya	20%	20%	0%	13%	5%	− 9%	11%	12%	0%
Mizoram	50%	52%	2%	45%	47%	2%	7%	6%	− 2%
Nagaland	17%	29%	12%	25%	29%	5%	10%	11%	1%
Orissa	15%	17%	2%	16%	17%	1%	20%	23%	4%
Puducherry	67%	68%	2%	74%	74%	0%	90%	93%	2%
Punjab	34%	37%	4%	37%	40%	2%	34%	32%	− -1%
Rajasthan	23%	25%	1%	26%	29%	3%	17%	18%	0%
Sikkim	11%	25%	14%	13%	18%	5%	3%	3%	0%
Tamil Nadu	44%	48%	4%	50%	53%	3%	40%	44%	4%
Telangana	32%	39%	7%	45%	52%	7%	30%	31%	1%
Tripura	17%	26%	9%	64%	63%	− 1%	21%	22%	1%
Uttar Pradesh	21%	22%	1%	25%	25%	1%	45%	55%	10%
Uttarakhand	26%	30%	5%	31%	35%	4%	23%	28%	5%
West Bengal	28%	32%	4%	47%	48%	1%	62%	69%	7%
India	28%	31%	3%	37%	39%	2%	38%	43%	5%

The rapid urbanization in Uttar Pradesh, Bihar, and West Bengal is characterized by continuing population growth in small settlements. As these settlements grew in population between 2001 and 2011, they are more likely to pass the population density threshold set by MAGPIE. MAGPIE does not use a threshold on agricultural employment share (as does the Census) or settlement population size (as does Indiapolis). Thus, MAGPIE categorically classifies small, dense settlements as urban. So, larger shares of the population in areas with this development pattern are considered urbanizing as more settlements pass the density threshold and as these relatively small settlements experience population growth. In contrast, the Census measures rapid urbanization in Kerala, because it considered only 26% of the population urban in 2001. Both Indiapolis and MAGPIE considered the majority of Kerala’s land area and population to be urbanized in 2001 already, so there is less potential for further urbanization.

## Discussion

We estimate that in 2011, India’s population was 43% urban, or 140 million more urban residents than estimated by the Census of India. MAGPIE places 18% of the total population and 48% of the urban population into very large, often polycentric urban agglomerations of greater than 10 million people while the Census of India considers the bulk of the urban population to be in mid-sized cities with populations between 100,000 and 1000,000. While the Census’ definition may characterize how these urban populations are administered, our method implies a much more spatially interconnected urban system, as well as divergent urbanization processes taking place in different regions of India. Like previous efforts to estimate India’s urbanization without reference to gender and employment categories (Denis et al. 2012), the implications are that substantial investment in services to support life in dense settlements will be required, whether or not the official figures classify populations living within networks of proximate, small and dense settlements as urban or rural. This problem is only partially addressed in Indian development planning. Perhaps the most visible related policy initiative is the National Rurban Mission begun in 2016, which aims to identify 300 “rurban” clusters of 20 villages each across the country and target each with a variety of local workforce training activities and urban amenities such as water, sanitation, public transport, and street lighting. However, much of the official documentation about the National Rurban Mission implies that the initiative is designed to facilitate the urbanization-in-place of villages, while many of the selected clusters are in fact spatially proximate to large urban agglomerations and could be considered peri-urban or suburban (Singh and Rahman 2018). Similarly, many of the state nodal development authorities in West Bengal, Bihar, and Uttar Pradesh, to the extent that they address development in *gram panchayats*, are generally targeted towards the fringes of urban agglomerations rather than networks of villages undergoing in situ densification independent of a large city. Some major exceptions include the Gangasagar Bakkhali Development Authority in West Bengal that covers much of the Hooghly River estuary (Gangasagar Bakhali Development Authority 2019); the Patharchapuri, Barkreswar, Furfura Sharif, and Tarapith Development Authorities in West Bengal covering small rural regions covering 30–100 km^2^ (Urban Development Branch 2019); and the Kerala Local Government Service Delivery Project, allocating resources to for governance, capacity building, and infrastructure to all local governments outside of the six largest cities in the state (Local Self Government Department 2019).

We also make a methodological contribution to the problem of urban system definition. When provided with population counts at a sufficient spatial granularity, MAGPIE can rapidly and easily delineate urban areas and measure their populations in a much less labor-intensive process than the locality-based methods of e-Geopolis (the global project of which Indiapolis is part). While our implementation here depends on dasymetric population disaggregation, this method could be applied to other population grid products (such as Landscan or GHS-POP) to construct urban hierarchies that account for non-contiguous urban interconnectivity.

MAGPIE has an added advantage of automatically determining the edge of the city without relying solely on the contiguity criterion. The intuition behind this method is that urban areas can be non-contiguous at the edges (Schnieder and Woodcock 2008) and this method allows for them to become part of the urban region. This is similar but not identical to US Census delineation of urban areas using a “hop and jump” criteria to account for discontinuous urbanization (Ratcliffe et al. 2016). One way to account for discontinuous urbanization is to merge urban areas that are within a certain distance from one another. However, if we simply merge urban areas that are within a distance threshold, then there will be situations where urban extents will have tendril/tail or hourglass-like forms due to sparse connections at the edges or between two large urban areas. Tendrils are observed when urban development is caused by linear infrastructure expansions such as highways. MAGPIE allows for these tails to separate urban clusters as there are only a few edges between them. This method also would merge two large urban regions into one, only when there is sufficient number of smaller polygons that are in close proximity to both. Furthermore, if many small urban areas are non-contiguous, by virtue of them being close to one another, they can form an urban cluster and could be treated as a single unit.

Urbanity is a continuum and the standard dichotomy between urban and rural is not adequate to characterize the human settlement patterns and their changes (Hugo et al. 2004; Wratten 1995). However, because the level of urbanization is considered a proxy for development, we argue that consistent characterizations of urban boundaries are useful. Hugo et al. (2004) argue that settlements ought to be measured on different dimensions, including size, concentration, and accessibility within the region. While our method accounts for the first two characteristics explicitly, we do not account for the access characteristics, which should be addressed in the future.

Another limitation of MAGPIE as currently implemented is an inconsistent agglomeration of peninsular or island settlements into surrounding urban areas from which they are separated by water features. This is illustrated by the high-density, low population identified agglomerations of Old Kochi, Willingdon Island, Ramanthuruth, and Bengre, all of which are part of the cities of Kochi or Mangalore (see lower three rows of Fig. 5).

Furthermore, our method of agglomerating urban settlements depends on the accuracy of the dasymetric disaggregation of census counts, which are only available to us, at the relatively coarse spatial unit. This could contribute to our method’s production of large urban agglomerations over areas that are traditionally considered rural, if densely populated. This could also contribute to error in the other direction, as our disaggregation may allocate population growth that actually occurred in concentrated cities throughout the district in which a given city is located. Other data products have become recently available at finer geographic scale that could have improved the results (e.g., Balk et al. 2019; Meiyappan et al. 2018). However, they also suffer from poor spatial precision of spatial units and reconciling them to create temporally consistent units is an arduous task. In any case, all gridded population estimates depend crucially upon the underlying official district-level geographies and counts. Some limitations related to modeling approach could also affect this estimation. As RF is a tree-based estimator, it is restricted by the range of training. The prediction using RF model trained on district-level population density and the zonal mean of covariates will have lesser range and heterogeneity than the RF model trained on actual pixel scale population counts and covariate values. In other words, as the variability among the district-level population density is used to model the variability inside the districts, it will lead to a less heterogeneous predictions with smaller variance. In this regard, the sensitivity of MAGPIE with the resolution of training census data needs to be evaluated. Forecasting future urbanization based on current non-linear relationships among the underlying covariates might be problematic. However, scenario-based forecasting that estimates future urbanization based on relationships among subsamples might provide some direction for future research. In addition to the errors associated with other environmental and remote sensing datasets, we ought to be mindful of this limitation.

## Conclusions

In this paper, we show that definitional differences and seemingly innocuous choices of thresholds matter a great deal for the delineation and categorization of urban settlements. Not only the population thresholds matter but also the density cut-offs are important in distinguishing urban from rural. The density thresholds also affect the contiguity and delineation of urban areas, which in turn affect the total population thresholds. Depending on the density cut-off, 35% to 57% of India's population lives in “urban” areas in 2011 (contrast with 31% estimated by the Census of India). Additionally, about 5–7 million people live in about 20,000 distinct small towns (< 5000 population) with relatively high density. Highly dense regions in the Gangetic plain are contiguous enough to form large agglomerations. Furthermore, because we do not rely on the political and jurisdictional boundaries and instead rely on a contiguity criterion, our estimates on the number of medium-sized towns (less than 100k population) are significantly lower than the Census or the Indiapolis estimates by Denis and Zérah (2017). Instead, these small towns are coalesced into much larger urban agglomerations, thus changing the conclusions that can be drawn about the type and extent of urbanization in India. The differing boundaries of urban areas and much larger agglomeration of small towns can be attributed to the accuracy of the gridded surface. Still, the contiguity-based criterion provides a meaningful way to compare the urban agglomerations.

We find that the results from our method also challenge the idea about the declining urbanization rate in India. While there may be strong political and governance reasons for large and dense “rural” areas to be classified as urban, they pose a problem for comparative statistics. Our contribution lies in the methodology to harmonize the differences and provide a consistent characterization of urban across large regions. This work can be extended in a few ways. One extension could be analyzing the sensitivity of this approach with the change in the scale of training data and by using other types of gridded population dataset products. Another possible extension could be modeling multiple contiguous countries together with a different economic status and analyzing the difference in urbanization. This work demonstrates the importance of seemingly benign and arcane definitional matters to the measurement of urbanization. Recognizing them would help us fashion institutions and jurisdictions that are better aligned to manage urban growth.

## Footnotes

1 Dijkstra and Poleman (2014) also use 15 persons per ha to define high-density urban cluster for Europe.

2 We tested the sensitivity of the distance threshold by using 200 and 250 m. They resulted in much larger contiguous urban areas and therefore are not reported here.
